# Quality of Life and Poor Oral Health: A Comparison of Postmenopausal Women

**DOI:** 10.3390/dj4040044

**Published:** 2016-11-24

**Authors:** Kristin A. Williams, Hebba Shamia, Christine DeBaz, Leena Palomo

**Affiliations:** 1 Department of Community Dentistry, School of Dental Medicine, Case Western Reserve University, 2124 Cornell Road, Cleveland, OH 44106, USA; kristin.williams@case.edu (K.A.W.); hebba.shamia@case.edu (H.S.); christine.debaz@case.edu (C.D.); 2 Department of Periodontics, School of Dental Medicine, Case Western Reserve University, 2124 Cornell Road, Cleveland, OH 44106, USA

**Keywords:** oral health, quality of life, postmenopausal women

## Abstract

Inter-relationships between traditional dental variables are becoming more evident in far reaching aspects of life, such as psychosocial interaction, self-esteem, overall health and even occupational performance. This study compares quality of life (QoL) in postmenopausal women (PMW) with poor oral health (POH) with QoL in PMW with good oral health. A total of 200 randomly recruited PMW received a dental evaluation and completed the Utian Quality of Life Survey. The participants were divided into POH and healthy groups based on a dental exam. Mean scores were calculated for each QoL item, domain and the overall summary score. For each of the four parameters for periodontitis diagnosis, periodontitis b s patients’ QoL outcomes were compared to those of healthy patients using a T-test with a threshold of significance at *p* < 0.05. QoL in all fields measured was significantly poorer in the POH patients compared to the healthy patients: occupational score (19.95 ± 5.35 vs. 27.56 ± 6.13), health score (18.02 ± 8.23 vs. 26.59 ± 6.45), emotional score (15.68 ± 10.22 vs. 21.15 ± 9.15), sexual score (6.2 ± 5.98 vs. 10.02 ± 5.35), and total score (60.21 ± 25.85 vs. 84.26 ± 22.35). This study finds that PMW with POH report significantly poorer quality of life. Clinicians caring for PMW should be aware that oral health impacts QoL and make appropriate referral decisions for patients’ dental care.

## 1. Introduction

The effect of oral health on quality of life (QoL) is emerging as a valuable area of investigation. The World Health Organization considers oral-health to have a far-reaching impact on QoL [1]. As dentists, we identify that a healthy smile has much to do with psychosocial interaction, self-esteem, and relationships. Comfortable functioning, free of pathology, has always been the goal of the clinical profession, but now this thought process is converging with evidence-based research [2].

However, inter-relationships between and among traditional variables, such as caries and periodontitis and pulpitis diagnosis, with the more current direction of patient-centered subjective QoL, has not yet been elucidated for different populations. Much of the dental research around QoL has centered on child and adolescent populations having developmental abnormalities such as clefts [3]. Investigations around caries prevention and control focus on access to care, in a backdrop of developing countries or economically challenged areas of the United States [4,5,6,7].

The relatively healthy, ambulatory patients, who make up the majority of dental practices have not been a focus.

On the other hand, investigations focused on the effect of esthetics on QoL tend to focus on the lower third of the face, not just the teeth and surrounding tissues, and have been dominated by medical journals. These studies shine a light on the far-reaching relevance of esthetics on the lives of postmenopausal women [8,9,10,11]. Although most of these take a closer look at soft tissues of the lips and extraoral tissues, investigations by plastic surgeons conclude that postmenopausal women specifically, are driven to facial esthetics focusing on the lower third of the face as opposed to other women who tend to focus esthetic demands on the skin and nose [12].

With women living longer and more vibrant lives than ever, investigating this as a specific population is useful.

Dental measurement instruments, such as the Oral Health Impact Profiles (OHIP), are used to measure oral- health-related QoL. However, OHIP, and other instruments like it, address functional limitations associated with acute presentations, such as difficulty pronouncing words, physical pain and disability. Mostly, patients suffering from chronic periodontitis or untreated decay do not present with such an obvious, acute presentation that would compromise speech or generate pain and disability. Esthetic complaints likewise impact psychosocial endpoints. Reports suggest that even in the absence of acute presentation, the dental condition impacts QoL as a more complex interaction; chronic conditions and esthetic complaints deviate from the norm, establishing a “new normal” which is a QoL compromise [13,14,15]. Similarly, dental interventions involving implants to replace lost teeth improve QoL [16].

Dental studies on oral disease management commonly examine the subject on the basis of pathogenesis, risk factors, treatment efficacy and outcome. These measurements involve clinical variables and not individual patient perspective as the focus of investigations. From 1950 to 2013, the majority of QoL papers and research models are based on “sick-role” related theories and do not readily embrace biopsychosocial theories of health. More recently, there has been a switch in this pattern. Outcomes such as esthetics, comfort etc., are subjective and are difficult to measure. As such, more objectively identifiable parameters around oral disease are being selected to make poor oral health and control groupings. The outcome measurement presumes that the effect of the more subjective outcomes will be identified by the questionnaire and then tied to the groupings. There is no precedent that the authors could find on a better way to create a dividing line between poor oral health and the control groups [17]. These dental focused instruments only tangentially address occupational, psychosocial and systemic health related endpoints. It stands to reason that any chronic low-grade disease produces a wide range of effects that over time would tax these dimensions of wellness. More importantly, the OHIP and other dentally focused instruments are not validated in postmenopausal women. QoL is understood to be imprecisely defined, and has misleading conclusions. How postmenopausal women with poor oral hygiene perceive QoL versus healthy controls is not reported. This study compares QoL in postmenopausal women with poor oral health with those with good oral health.

## 2. Methods

A total of 200 randomly selected subjects from the Case Western Reserve University/Cleveland Clinic Postmenopausal Wellness Collaboration (CCCPW) participated in this cross-sectional investigation. Informed consent was obtained from all participants. The CCCPW database includes over 900 participant samples with comprehensive oral examination dates from January 2002 to October 2014. In this database, all participants are defined as postmenopausal by either natural or surgical/medical means.

Each participant received a comprehensive oral exam, as defined by the American Dental Association, including a comprehensive periodontal charting, as defined by the American Academy of Periodontology from a panel of board certified periodontists [18,19]. Examiners are calibrated annually to an electronic force controlled probe. Inter- and intra- examiner calibration statistics are shown to have no significant difference (*p* < 0.05) in Table 1. A comprehensive oral examination includes health history, a review of prescriptions the patient is using, extra-oral head and neck exam, intra-oral examination including a review of all restorations, screening for decay, and radiographic review. The periodontal exam includes periodontal probing using a North Carolina Probe with millimeter markings such that six sites per tooth are probed with 25 N force; third molars are excluded, recording the percentage of sites which have bleeding on probing; locations of the gingival margin and mucogingival junction, and plaque score. Furcation involvement is noted if there is any. Based on exam outcomes, participants were given the designation of poor oral health if they met at least one of the following criteria: (1) whole mouth mean clinical attachment loss (CAL) ≥3 mm which denotes the loss of structures, periodontal ligament, cementum and alveolar bone, this is a direct measure of periodontal disease; (2) single highest probing depth in the mouth ≥5 mm; the most severe pocketing without the washout effect of a mean; (3) >5 surfaces of untreated decay; (4) any site of decay to the extent of radiographically apparent pulpal involvement.

### 2.1. Compliance with Ethical Standards

The authors have no conflict of interest to disclose. All procedures performed in this study are in accordance with the ethical standards of the institutional research committee, which is, in turn, in accordance with the 1964 Helsinki declaration and its later amendments or comparable ethical standards. The investigation was reviewed and Institutional Review Board (IRB) approved (number 2014-814) by the Case Western Reserve University IRB. Informed consent was provided by all participants during the questionnaire phase of the study.

### 2.2. Utian Quality of Life Survey

Participants completed the Utian QoL questionnaire, with a scale indicating the extent to which the test question is experienced. The items are combined into domains including occupational, health, emotional, and sexual, resulting in a total overall summary score (additive method). This survey has been validated and shown to be reliable and reproducible [20].

### 2.3. Analysis

For each of the four criteria designated for poor oral health, QoL outcomes of the poor oral health group were compared to those of the healthy group using T-test with a threshold of significance at *p* < 0.05. Logistic regression for related variables (Body Mass Index (BMI), low bone mass, tobacco use, hormone replacement, bone sparing medications and diabetes) are expressed in terms of *p* value.

## 3. Results

A total of 110 participants met one of the four designated criteria for poor oral health. A breakdown of each criterion is found in Table 2. Ten poor oral health group patients had more than one qualifying criterion. There is no significant difference in age between the group with poor oral health and the control. Participant demographics are noted in Table 3. Participants differed for characteristics including diabetes, use of bone sparing medications, tobacco and high BMI. It is of note that some participants used more than one bone sparing medication. Therefore, the sum of the fields in the bone sparing medications column of Table 3 adds up to more than the number of participants. Similarly, four participants did not report tobacco use and that field reflects this lack of participation. Table 4 reflects dental characteristics of the participants. Notably, probe depth was not significantly associated with plaque score, or frequency of visits and these dental characteristics are not significantly different between groups.

Table 5 compares QoL scores in the poor oral health group versus the control across the four domains and overall. The Poor Oral Health (POH) group has a significantly different occupational, health, emotional and sexual score. The overall score is also significantly different between the groups.

## 4. Discussion

Findings show that QoL is worse in the poor oral health group versus the healthy controls in the dimensions of occupational, health, emotional, and sexual scores. Not surprisingly, then, the total score also shows that QoL is worse in the poor oral health group. This is in line with the World Health Organization (WHO)’s decision to portray QoL images beyond just pain-free living, but rather with esthetic images showing attractive smiles as its image of enhanced well-being. The American Dental Association echoes this sentiment as it cites an emerging sense of consumerism among the users of dental care services; people are becoming more astute purchasers of health care and seek to retain their teeth using mostly out-of-pocket spending [21]. That is to say, dental services aim at restoring comfort, function and esthetics. In light of this angle, it is not surprising that our survey finds that those who lack such wellness are disadvantaged when QoL is reflected in seemingly far-off domains, such as occupational, health, emotional and sexual scores. It is well documented that oral health is potentially linked to many systemic diseases, such as diabetes, heart disease and stroke in addition to poor pregnancy outcomes [22,23,24]. Being overweight and obesity lie at the intersection of these conditions. It is not surprising that the results show significant differences in low, medium and high BMI participants.

Since the underlying mechanisms of the oral systemic disease link are foundational, the authors of this study interpret that they have an impact on more far reaching areas of function. Poor oral health can affect occupational QoL though the presence of chronic pain, persistent discomfort to a point which would affect performance and satisfaction with work. The American Academy of Pain Medicine notes that the burden of pain is felt in various components of everyday life and quantifies productivity losses as a consequence of it [25]. Similarly, perceived esthetic compromise is reportedly associated with anxiety and depression in children and adolescents; it is easily extrapolated to adults, especially in light of long-term studies of patients with craniofacial abnormalities which elucidate results of lower facial esthetics in adulthood [26,27]. It is therefore understandable how poor oral health could contribute to a social self-consciousness, having its endpoints in sexual and emotional fields.

Post hoc analysis shows some significant odds ratios for poor oral health. Tobacco users have a 31% (CI 95%; 0.71, 0.58) greater rate of poor oral health and diabetics are at a 5.2 (CI 5.21%; 1.75, 15.45) times greater risk of having poor oral health. This is consistent with the consensus in dental literature that tobacco causes various oral health diseases, and that diabetes is a risk for oral diseases [28,29]. However, in a departure from the majority of dental literature, race does not significantly affect the odds of poor oral health [30,31,32]. One possible explanation for this departure is that in this study the participants are drawn from the CCCPW, a database where all participants have access to both medical and dental care. The participant profile does not reflect a random sampling of the community, but rather a more limited socio-economic group that has access to care. It also supports the assertion made recently by Burgette et al that oral health literacy plays a key role and suggests that if socioeconomics is controlled, race alone does not confer poor oral health risk [33].

The odds of poor oral health in postmenopausal women who use bone sparing medications are 21% (CI 95% 0.11, 0.41). Differing dental health complications have been linked to different medications, for example, bleeding gums have been associated with hormones, and osteonecrosis has been associated with Intravenous (IV) bisphosphonates [34,35]. The differing effect of each of the different bone sparing medications is beyond the scope of the current study, but is an interesting focus for future research, focused on women.

This study is provocative because it focuses on a population which is not well studied in dentistry: postmenopausal women. According to Forbes magazine, women, particularly in this age group, are the world’s most powerful consumers. It is even suggested that women have been a traditionally blind spot for businesses, and the demographic should be studied specifically, as one would a foreign market [36]. Future studies are needed to compare the effect of race, education and socio-economic status within this population.

This study is also unique because it looks beyond these measures to the correlates in psychosocial arenas provided by an instrument which is validated in this specific population.

A recent paper critically examined the different models for oral health, representing oral health related QoL, and found that despite a multitude of representations of concepts, dental research has remained linear in portraying the consequences of disease as the absence of disease, dysfunction and disability [37].

## 5. Conclusions

This study finds that postmenopausal women with poor oral health, report significantly poorer quality of life. This is reflected across dimensions of occupational, health, emotional, and sexual health. Since poor oral health has broad impacts, clinicians caring for postmenopausal women should be aware of the importance of oral health and make appropriate decisions to refer to a periodontist for patients’ care needs when it is indicated.

## Figures and Tables

**Table 1:** Examiner Calibration Data ANOVA is used to identify no significant differences between a panel of examiners. T-test is used to identify no significant differences between time 1 and time 2 of each of the examiners.

Location	Inter-Examiner	Intra-Examiner 1	Intra-Examiner 2	Intra-Examiner 3
Mesiobuccal	0.03	0.04	0.02	<0.001
Buccal	<0.001	<0.001	0.03	<0.001
Distobuccal	0.02	0.02	0.04	0.02
Mesiolingual	0.02	0.03	0.03	0.02
Lingual	<0.001	<0.001	0.03	<0.001
Distolingual	0.03	0.04	0.04	0.04

**Table 2:** Criteria breakdown in the poor oral health group based on oral exam (N).

Criteria Breakdown	No. of Participants
Whole mouth mean clinical attachment loss >3 mm	33
Single highest probing depth in the mouth ≥5 mm	49
>5 surfaces of untreated decay	12
Any site of decay to the extent of radiographically apparent pulpal involvement.	26
Poor Oral Health (sum of the above)	120

**Table 3:** Demographics divided according to poor oral health and control.

Characteristic		Poor Oral Health	Control	*n*	*p*-Value
Average Age	65 ± 7.1 years	62.3 ± 5.5	67.7 ± 8.6	200	
Race/Ethnicity					0.86
	White	55	40	95	
	Black	57	38	96	
	Other	8	2	9	
Body Mass Index (BMI)					0.002 *
	BMI < 19	6	39	45	
	20 < BMI < 29	55	33	88	
	BMI > 30	49	18	61	
Low Bone Mass T < 2.0					0.137
	Low Bone Mass (LBM)	72	38	110	
	Normal	38	52	90	
Tobacco use					0.0005 *
	Never	21	54	75	
	Former	80	35	115	
	Current	6	0	6	
	Did not report	3	1	4	
Hormone Replacement					0.63
	Never	19	20	39	
	Former	86	67	153	
	Current	5	3	8	
Bone Sparing Medications					0.0001 *
	Oral Bisphosphonate	18	69	87	
	Intravenous (IV) Bisphosphonate	2	9	11	
	SERM	1	6	7	
	RANKL Inhibitor	0	3	3	
	None	89	3	92	
Diabetes					0.0012 *
	Yes (HbA1c ≥ 8)	41	13	54	
					

* denotes significant difference.

**Table 4:** Dental characteristics. Probe depth was not significantly associated with plaque score, frequency of visits, nor were these dental characteristics different between groups.

	Poor Oral Health		Control	
Measurement	*r* Value	*p* Value	*r* Value	*p* Value
**Probe Depth**	0.153	0.418	0.28	0.882
**Percentage of Broken Appointments**	0.54	0.612	0.35	0.534
**Plaque score**	−0.051	0.789	−0.41	0.24

**Table 5:** Quality of life scores in the poor oral health group versus the control.

	Mean (Standard Deviation)	*p*-Value
**Occupational score**		
Control	27.56 ± 6.13	
Poor Oral Health (POH)	9.95 ± 5.35	
		0.000 *
**Health score**		
Control	26.59 ± 6.45	
POH	18.02 ± 8.23	
		0.000 *
**Emotional score**		
Control	21.15 ± 9.15	
POH	15.68 ± 10.22	
		0.0001 *
**Sexual score**		
Control	10.02 ± 5.35	
POH	6.2 ± 5.98	
		0.0001 *
**Total score**		
	84.26 ± 22.35	
	60.21 ± 25.85	
		0.000 *

## References

[1] Petersen P.E. (2003). The World Oral Health Report 2003: Continuous improvement of oral health in the 21st century—The approach of the WHO Global Oral health Programme. Community Dent. Oral Epidemiol..

[2] Department of Health and Human Services (2000). Oral Health in America: A Report of the Surgeon General.

[3] Sischo L., Broder H.L. (2011). Oral Health-related Quality of Life. J. Dent. Res..

[4] Gambhir R.S., Brar P., Singh G., Sofat A., Karkar H. (2013). Utilization of dental care: An Indian outlook. J. Nat. Sci. Biol. Med..

[5] Ganavadiya R., Chandrashekar B., Goel P., Hongal S.G., Jain M. (2014). Mobile and portable dental services catering to the basic oral health needs of the underserved population in developing countries: A proposed model. Ann. Med. Health Sci. Res..

[6] Ha J.E., Heo Y.J., Jin B.H., Paik D.I., Bae K.H. (2012). The impact of the National Denture Service on oral health-related quality of life among poor elders. J. Oral Rehabil..

[7] Divaris K., Lee J.Y., Baker A.D., Vann W.F. (2011). The relationship of oral health literacy with oral health related quality of life in a multi-racial sample of low income female care givers. Health Qual. Life Outcomes.

[8] Dutt P., Chaudhary S., Kumar P. (2013). Oral health and menopause: A comprehensive review on current knowledge and associated dental management. Ann. Med. Health Sci. Res..

[9] McCann A.L., Bonci L. (2001). Maintaining woman’s oral health. Dent. Clin. N. Am..

[10] Buencamino M.C., Palomo L., Thacker H.L. (2009). How menopause affects oral health and what we can do about it. Clevel. Clin. J. Med..

[11] Suri V., Suri V. (2014). Menopause and oral health. J. Midlife Health.

[12] Bowler P.J. (2009). Impact on facial rejuvenation with dermatological preparations. J. Clin. Interv. Aging.

[13] DeBaz C., Shamia H., Hahn J., Mithani S., Sadeghi G., Palomo L. (2015). Periodontitis Impact Quality of life in postmenopausal women. Climacteric.

[14] McGrath C., Comfort M.B., Lo E.C., Luy Y. (2003). Can third molar surgery improve quality of life? A 6-month cohort study. J. Oral Maxillofac. Surg..

[15] Jansson H., Wahlin A., Johansson V., Akerman S., Lundegren N., Isberg P.E., Norderyd O. (2014). Impact of Periodontal Disease Experience on Oral Health-Related Quality of Life. J. Periodontol..

[16] DeBaz C., Hahn J., Lang L., Palomo L. (2015). Dental Implant supported Restorations Improve Quality of Life in Osteoporotic women. Int. J. Dent..

[17] Utian W.H., Janata J.W., Kingsberg S.A., Schluchter M., Hamilton J.C. (2002). The Utian Quality of Life (UQOL) Scale: development & validation of an instrument to quantify quality of life through and beyond menopause. Menopause.

[18] (1995). Evaluation: Patient Requiring a Comprehensive Oral Evaluation. http://www.ada.org/en/science-research/dental-practice-parameters/evaluation-patient-requiring-a-comprehensive-oral-evaluation.

[19] United States Air Force School of Aerospace Medicine (1963). A Screening Examination for Detection of Gingival and Periodontal Breakdown and Local Irritants.

[20] Abay H., Kaplan S. (2016). Validation and Reliability of the Turkish Utian Quality-of-Life Scale in postmenopausal women. Menopause.

[21] Taylor J.J., Preshaw P.M., Lalla E. (2013). A review of the evidence for pathogenic mechanisms that may link periodontitis and diabetes. J. Periodontal..

[22] Schenkein H.A., Loos B.G. (2013). Inflammatory mechanisms link periodontal diseases to cardiovascular diseases. J. Periodontal..

[23] Madianos P.N., Bobetsis Y.A., Offenbacher S. (2013). Adverse pregnancy outcomes (APOs) and periodontal disease pathogenic mechanisms. J. Periodontal..

[24] The Committee on Advancing Pain Research Care and Education (2011). Relieving Pain in America, a Blueprint for Transforming Prevention, Care, Education and Research.

[25] Barbosa T.S., Gavião M.B.D., Castelo P.M., Leme M.S. (2016). Factors Associated with Oral Health-related Quality of Life in Children and Preadolescents: A Cross-sectional Study. Oral Health Prev. Dent..

[26] Albers A.E., Reichelt A.C., Nolst-Trenite G.J., Menger D.J. (2016). Feeling Normal? Long-Term Follow-up of Patients with a cleft Lip-Palate after Rhinoplasty with the Derriford Appearance Scale. Facial Plast. Surg..

[27] The Research, Science and Therapy Committee of The American Academy of Periodontology (1999). Position Paper: Tobacco Use and the Periodontal Patient. J. Periodontal..

[28] Oliver R.C., Torvonin T. (1994). Diabetes—A Risk Factor for Periodontists in Adults. J. Periodontal..

[29] Kelesidis N. (2014). A racial comparison of sociocultural factors and oral health perceptions. J. Dent. Hyg..

[30] Shelley D., Russell S., Parikh N.S., Fahs M. (2011). Ethnic disparities in self-reported oral health status and access to care among older adults in NYC. J. Urban Health.

[31] Albandar J.M., Kingman A. (1999). Gingival Recession, Gingival Bleeding and Dental Calculus in Adults 30 Years and Older in the United States, 1988–1994. J. Periodontal..

[32] Horner-Johnson W., Dobbertin K., Beilstein-Wedel E. (2015). Disparities in dental care associated with disability and race and ethnicity. J. Am. Dent. Assoc..

[33] Burgette J.M., Lee J.Y., Baker A.D., Vann W.F. (2016). Is Dental Utilization Associated with Oral Health Literacy. J. Dent. Res..

[34] Knight E.T., Liu J., Seymour G.J., Faggion C.M., Cullinan M.P. (2016). Risk factors that may modify the innate and adaptive immune responses in periodontal diseases. Periodontal 2000.

[35] American Association of Oral and Maxillofacial Surgeons (2009). Advisory Task Force on Bisphosphonate-Related Ostenonecrosis of the Jaws. J. Oral Maxillofac. Surg..

[36] Brennan B. (2015). Tope 10 Things Everyone Should Know about Women Consumers.

[37] Brondani M.A., MacEntee M.I. (2014). Thirty years of portraying oral health through models: What have we accomplished in oral health-related quality of life research. Qual. Life Res..

